# Numerical and Experimental Investigation of Orientation Deviation in Shear Band in Grain-Oriented Silicon Steel

**DOI:** 10.3390/ma18102229

**Published:** 2025-05-12

**Authors:** Sihao Chen, Fang Zhang, Yuhui Sha, Xi Chen, Liang Zuo

**Affiliations:** 1 Key Laboratory for Anisotropy and Texture of Materials (Ministry of Education), Northeastern University, Shenyang 110819, China; chensihao1992@outlook.com (S.C.); chenxi@dlpu.edu.cn (X.C.); yhsha@mail.neu.edu.cn (Y.S.); lzuo@mail.neu.edu.cn (L.Z.); 2 School of Mechanical Engineering and Automation, Dalian Polytechnic University, Dalian 116034, China

**Keywords:** shear band, Goss orientation, orientation rotation, crystal plasticity, grain-oriented silicon steel

## Abstract

As a critical factor for the magnetic properties of grain-oriented silicon steel, the orientation accuracy of shear bands is closely related to the matrix orientation deviation from {111}<112>. This work investigates the orientation rotation of shear bands in {111}<112> matrices with various types of deviation during cold rolling, using a visco-plastic self-consistent model that incorporates a two-dimensional inclined angle of the shear band dependent on matrix orientation. When the matrix orientation deviates from {111}<112> along φ_1_, φ_2_, or both axes, the φ_1_ deviation of the shear band decreases, and the φ_2_ deviation is larger than φ_1_. Compared with a uniaxially deviated {111}<112> matrix, a biaxially deviated matrix along φ_1_ and φ_2_ axes produces a higher shear band deviation from Goss due to the increased φ_2_ deviation. This suggests that improving the orientation accuracy of the shear band is necessary to decrease the matrix deviation from {111}<112> in the φ_1_ and especially φ_2_ axes.

## 1. Introduction

Grain-oriented silicon steel is an important soft magnetic material primarily used in transformer cores. Its exceptional magnetic properties stem from the development of sharp Goss texture ({110}<001>, in which {110} parallels the normal direction and <001> parallels the rolling direction) after secondary recrystallization, characterized by <001> easy magnetization direction along the rolling direction (RD) [1,2,3]. Shear bands, locally strained thin regions during cold rolling, serve as preferential nucleation sites for recrystallized Goss grains [4,5,6,7]. Consequently, the orientation deviation of the shear band from Goss is critical for texture control of grain-oriented silicon steel.

Shear bands are mainly distributed in {111}<112> deformed matrix in silicon steel; thus, the matrix deviation from {111}<112> affects the orientation of the shear band and recrystallized grains. It is found that the accurate Goss shear band forms in the exact {111}<112> matrix with an inclined angle of 29°–36° to RD [8,9,10]. The orientation of the shear band rotates around RD//<001> when the {111}<112> deformed matrix rotates around <001> axis [11]. As the deviation angle of {111}<112> matrix around the normal direction (ND)/<111> axis increases, the recrystallized Goss texture weakens and gradually rotates toward the cubic texture [12,13,14]. The orientation accuracy and intensity of Goss texture decrease with the increasing diffusion angle of {111}<112> matrix [15,16,17]. It is therefore essential to systematically investigate how the deviation of {111}<112> matrix affects the deviation angle of the shear band.

Crystal plasticity simulation has been used to explore the orientation relationship between the deformed matrix and shear band in silicon steel by different methods with fixed inclination angles. Y. H. Sha reported the orientation of the shear band depending on the matrix by crystal plastic finite element method with a shear band system of fixed inclination angle [10]. Shear bands in exact {111}<112> matrix exhibit Goss orientation in the simulation using a visco-plastic self-consistent (VPSC) model by incorporating the experimentally observed inclined angle of the shear band [18]. The VPSC model is a mean-field crystal plasticity model with self-consistent schemes to account for the interaction between orientations [19,20]. Actually, the deviated {111}<112> matrix can influence the inclined angle and the orientation rotation path of the shear band [21]. However, the inclined angle and orientation of the shear band in deviated {111}<112> matrix have not been quantitatively analyzed.

In this work, shear band orientation in {111}<112> matrix with various types of deviation angle during cold rolling of grain-oriented silicon steel was investigated using the VPSC model and experimental measurement. The new 2D inclined angle of the shear band, which is dependent on matrix orientation, was incorporated in the VPSC model. The orientation rotation rate in the shear band was further calculated to analyze the deviation angle variation of the shear band. This study provides a theoretical basis for understanding the deviation origin of shear band orientation in grain-oriented silicon steel.

## 2. Simulation Method

### 2.1. VPSC Model

(1) Kinematics

Based on the theory of Asaro and Rice, deformation gradient can be divided into two parts according to the linear decomposition: elastic deformation gradient and plastic deformation gradient [22]. The elastic deformation part includes lattice distortion and crystal rotation, and the plastic deformation part is accomplished by dislocation slip. The deformation velocity gradient L can be decomposed into elastic and plastic components:

(1) $$L\;=\dot{F}\cdot F^{-1\;}=L^{e}+L^{P}$$

The slip system α can be represented by slip direction vector $s_{0}^{\left(\alpha \right)}$ and slip plane normal vector $m_{0}^{\left(\alpha \right)}$. Both vectors are unit vectors and satisfy geometric orthogonality:

(2) $$s_{0}^{\left(\alpha \right)}\cdot m_{0}^{\left(\alpha \right)}=0$$

The plastic deformation velocity gradient can be expressed as:

(3) $$L^{P}=D^{P}+W^{P}=\sum_{\alpha =1}^{K}({\dot{\gamma }}^{\left(\alpha \right)}s_{0}^{\left(\alpha \right)}⨂m_{0}^{\left(\alpha \right)})$$

(4) $$D^{P}=\left(L^{P}+L^{\mathrm{PT}}\right)/2=\sum_{\alpha =1}^{K}P^{\left(\alpha \right)}{\dot{\gamma }}^{\left(\alpha \right)}$$

(5) $$W^{P}=\left(L^{P}-L^{\mathrm{PT}}\right)/2=\sum_{\alpha =1}^{K}K^{\left(\alpha \right)}{\dot{\gamma }}^{\left(\alpha \right)}$$

where ${\dot{\gamma }}^{\left(\alpha \right)}$ denotes the shear strain of the *α*th slip system, and *K* denotes the total number of slip systems. P^(*α*)^ and K^(*α*)^ can be expressed as:

(6) $$P^{\left(\alpha \right)}=\left(s^{\left(\alpha \right)}{⨂m}^{\left(\alpha \right)}+m^{\left(\alpha \right)}{⨂s}^{\left(\alpha \right)}\right)/2$$

(7) $$K^{\left(\alpha \right)}=\left(s^{\left(\alpha \right)}{⨂m}^{\left(\alpha \right)}-m^{\left(\alpha \right)}{⨂s}^{\left(\alpha \right)}\right)/2$$

(2) Slip rate

According to Schmid’s law [23], the slip system is activated when the resolved shear stress τ^α^ is greater than a critical value, and the slip rate ${\dot{\gamma }}^{\alpha }$ of the αth slip system is related to the resolved shear stress τ^α^ as follows:

(8) $${\dot{\gamma }}^{\alpha }{=\dot{\gamma }}_{0}{\left|\frac{\tau^{\alpha }}{\tau_{c}^{\alpha }}\right|}^{n}\mathrm{sgn}(\tau^{\alpha })$$

${\dot{\gamma }}_{0}$ is the reference slip rate, n is the inverse of strain rate sensitivity factor, $\tau_{c}^{\alpha }$ is the critical resolved shear stress. $\mathrm{sgn}(\tau^{\alpha })$ is a symbolic function, sgn(x)=1 when x ≥ 0, and sgn(x)=-1 when x < 0.

(3) Strain hardening

The method developed by S.R. Kalidindi et al. [24,25] and Y. Zhou et al. [26] is used. Strain hardening parameters are shown in Equations (9) and (10):

(9) $${\dot{\tau }}_{c}^{\alpha }=\sum_{\beta =1}^{N}H^{\alpha \beta }\left|{\dot{\gamma }}^{\beta }\right|$$

where α, β = 1, 2, 3......N. ${\dot{\tau }}_{c}^{\alpha }$ is the strength of the αth slip system, ${\dot{\gamma }}^{\beta }$ is the slip rate of the βth slip system, N is the number of slip systems. $H^{\alpha \beta }$ is the self-hardening and latent-hardening matrices. The latter is defined as follows:

(10) $$H^{\alpha \beta }{=q}^{\alpha \beta }h_{0}{\left(1-\frac{\tau_{c}^{\alpha }}{\tau_{s}}\right)}^{a}$$

where h_0_ and a are hardening parameters and τ_s_ is the saturation shear stress. q^αβ^ is the asymmetric hardening matrix representing the interaction between slip systems. The elements of this hardening matrix are determined based on four possible geometrical configurations of the αth and βth slip systems: co-planar slip (q_1_), co-linear slip (q_2_), perpendicular slip (q_3_), and the same coefficients assigned to all other configurations (q_4_). {110}<111> and {112}<111> slip systems are considered with initial shear strengths of 220 MPa and 240 MPa, respectively [27,28,29,30]. By a fitting process [31], the following hardening parameter values are identified for silicon steel: h_0_ = 244 MPa, a = 0.48, τ_s_= 1137 MPa, q_1_ = 1.0, q_2_ = 1.5, q_3_ = 2.0, q_4_ = 1.5. ATEX software is used to calculate this VPSC model (version 4.14, ATEX-Université de Lorraine, Metz, France) [32].

### 2.2. Velocity Gradient Tensor of Shear Band

Previous studies have identified shear band formation as a softening mechanism, where shear bands develop along a specific geometric direction for a matrix orientations [33]. Shear bands were observed in {111}<112> deformed matrix at an inclined angle of approximately 35°, governed by the {110} secondary slip plane [9].

**Figure 1:**
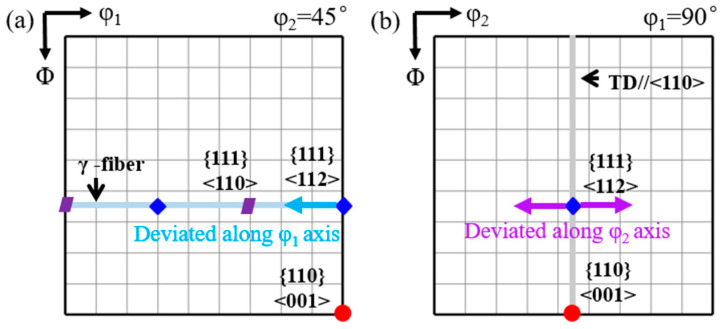
Schematic diagram of {111}<112> matrix deviated along (**a**) φ_1_ and (**b**) φ_2_ axis in constant ODF sections.

**Figure 2:**
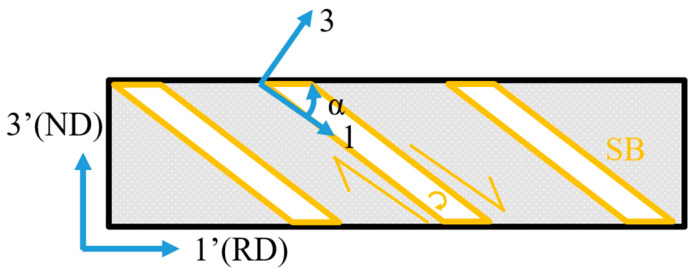
Schematic diagram of shear band inclined angle.

Figure 1 presents the schematic diagram of deviated {111}<112> matrix in φ_2_ = 45° and φ_1_ = 90° sections of orientation distribution function (ODF). Rotation of {111}<112> around ND//<111> corresponds to a deviation along the φ_1_ axis, while rotation around <001> corresponds to a deviation along the φ_2_ axis. Similarly, rotation of Goss orientation around ND//<110> is equivalent to the deviation along the φ_1_ axis, whereas the rotation around RD//<001> corresponds to the deviation along the φ_2_ axis. Figure 2 illustrates the geometrical relationship between the shear band and matrix, where the inclined angle θ of the shear band is determined as {110} secondary slip plane.

Based on the simple shear strain in the shear band coordinate system, the velocity gradient tensor of the shear band in the sample coordinate system is expressed as:

(11) $$L=\left[\begin{array}{ccc} \cos\alpha \sin\alpha & 0 & \cos^{2}\alpha \\ 0 & 0 & 0\\ -\sin^{2}\alpha & 0 & -\cos\alpha \sin\alpha \end{array}\right]$$

where α is the angle between the shear band plane and RD.

### 2.3. Calculation Scheme

**Figure 3:**
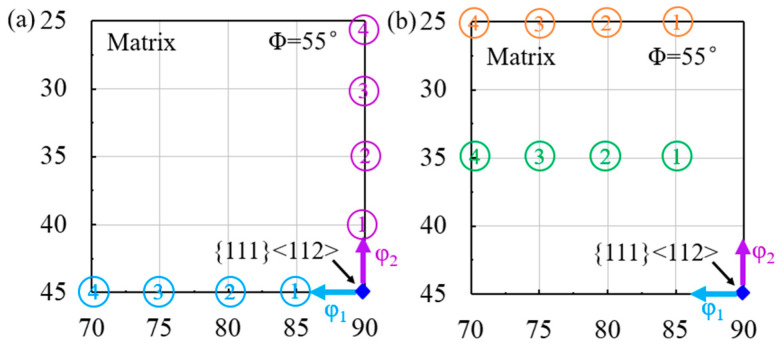
Schematic diagram of (**a**) uniaxially and (**b**) biaxially deviated {111}<112> matrix.

The deviated {111}<112> matrix is categorized into uniaxial and biaxial deviation in Euler space, as shown in Figure 3. The {111}<112> matrix that deviates along the φ_1_ axis (blue) or the φ_2_ axis (purple) by 5°, 10°, 15° and 20° is selected as the uniaxial deviation matrix. The {111}<112> matrix with biaxial deviation is selected as 10° (green) or 20° (orange) along the φ_2_ axis with 5°, 10°, 15°, and 20° along the φ_1_ axis. Furthermore, the orientation rotation rate of the shear band, the Euler angle change per unit strain, is calculated for different deviated {111}<112> matrices.

## 3. Results

### 3.1. Uniaxial Deviation of {111}<112> Matrix

#### 3.1.1. Deviation Along φ_1_ Axis

**Figure 4:**
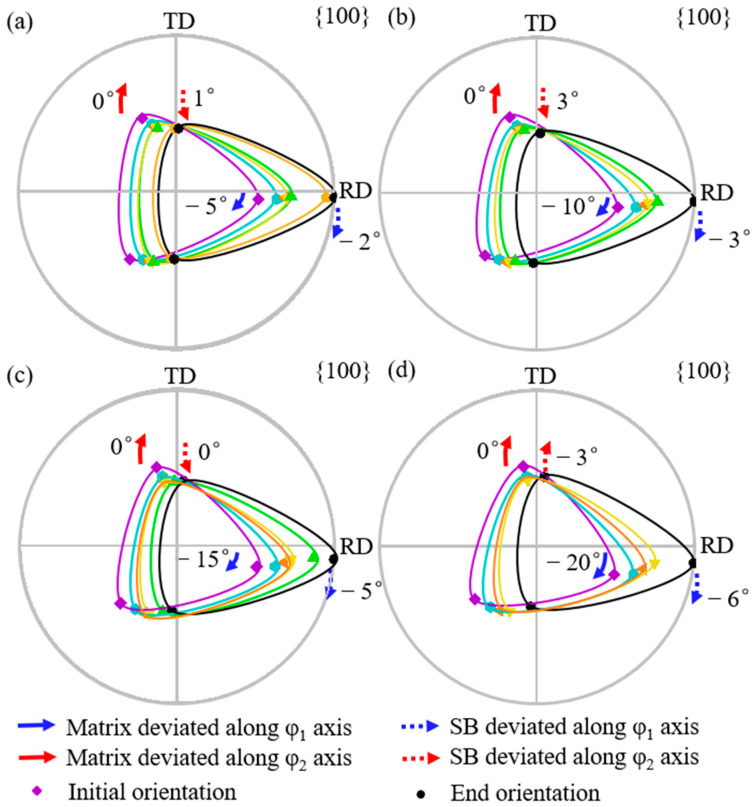
Simulated orientation rotation process of shear band in {111}<112> matrix deviated along φ_1_ axis by 5°, 10°, 15°, and 20° (**a**–**d**).

Figure 4 shows the simulated orientation rotation path morphologies of the shear band with the {111}<112> matrix deviated by 5° to 20° (a–d) along the φ_1_ axis. Solid arrows indicate the angle by which the matrix orientation deviates from exact {111}<112>, while dashed arrows show the angle by which the shear band orientation deviates from exact Goss. Blue arrows represent the deviation along the φ_1_ axis, and red arrows represent deviation along the φ_2_ axis. Counterclockwise rotation is designated as positive. From Figure 4a to Figure 4d, the deviation angle of the matrix orientation along the φ_1_ axis increases from 5° to 20°, while the deviation angle of the shear band orientation along the φ_1_ axis increases from 2° to 6°. The deviation angle of the shear band along the φ_1_ axis is always smaller than that of the matrix orientation. The deviation angle of the shear band along the φ_2_ axis first increases counterclockwise from 1° to 3° and then clockwise to −3°. In all cases, the shear band orientation exhibits a larger deviation along the φ_1_ axis compared with the φ_2_ axis.

**Figure 5:**
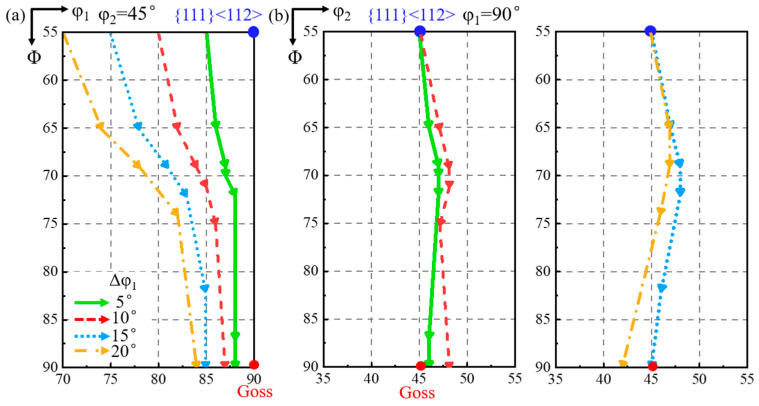
The rotation path projection of shear band orientation on constant (**a**) φ_2_ = 45° and (**b**) φ_1_ = 90° ODF sections with the matrix deviated along φ_1_ axis.

Figure 5 shows the rotation path projection of shear band orientation with {111}<112> matrix deviated along the φ_1_ axis. Each arrow represents the rotation under identical strain. The deviation angle of the shear band along φ_1_ decreases during rotation, while that along the φ_2_ axis first increases and then decreases. A larger deviation angle of the matrix produces a more significant decrease in φ_1_ and φ_2_ deviation angles of the shear band, respectively.

#### 3.1.2. Deviation Along φ_2_ Axis

**Figure 6:**
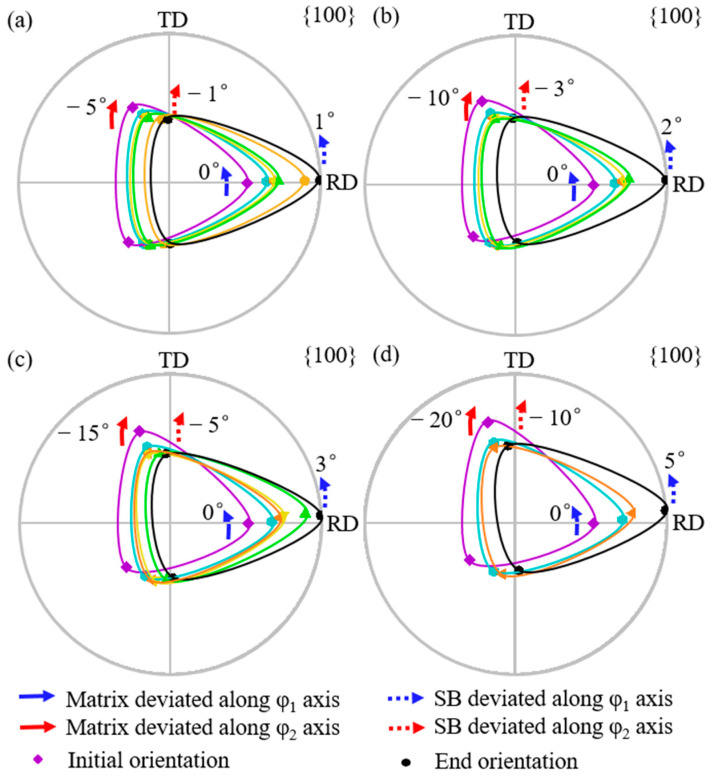
Simulated orientation rotation path of shear band in {111}<112> matrix deviated along φ_2_ axis by 5°, 10°, 15°, and 20° (**a**–**d**).

Figure 6 shows the simulated orientation rotation path of the shear band when {111}<112> matrix is deviated by 5° to 20° (a–d) along the φ_2_ axis. From Figure 6a to Figure 6d, with the deviation angle of the matrix along the φ_2_ axis increasing from 5° to 20°, the shear band deviation along the φ_2_ axis increases from 1° to 10°. The shear band deviation along the φ_2_ axis is smaller than the matrix as observed experimentally [11]. In addition, the deviation of shear band orientation along the φ_2_ axis is greater than or similar to that along the φ_1_ axis.

**Figure 7:**
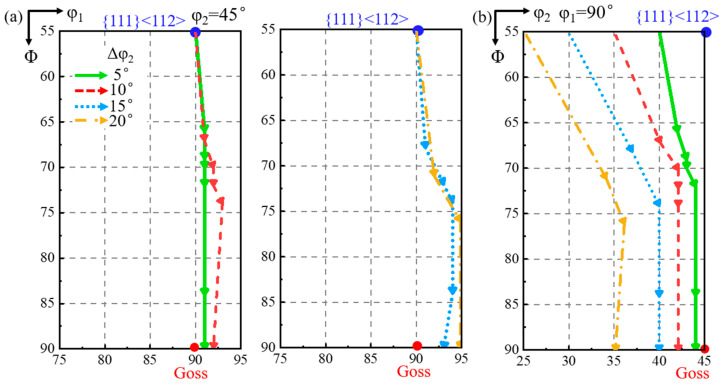
The projection of orientation rotation path of shear band for matrix deviated along φ_2_ axis on constant (**a**) φ_2_ = 45° and (**b**) φ_1_ = 90° ODF sections.

Figure 7 shows the projection of the orientation rotation path of the shear band when {111}<112> matrix is deviated along the φ_2_ axis. As shown in Figure 7a, the φ_1_ deviation of the shear band initially increases and then decreases slightly during rotation. The larger the matrix deviation along the φ_2_ axis, the greater the increase in the φ_1_ deviation of the shear band. According to Figure 7b, the φ_2_ deviation of the shear band decreases throughout the rotation process. A larger matrix deviation leads to a greater φ_1_ deviation, but with a more significant decrease in φ_2_ deviation.

### 3.2. Biaxial Deviation of {111}<112> Matrix

#### 3.2.1. 10° Deviation Along φ_2_ Axis

**Figure 8:**
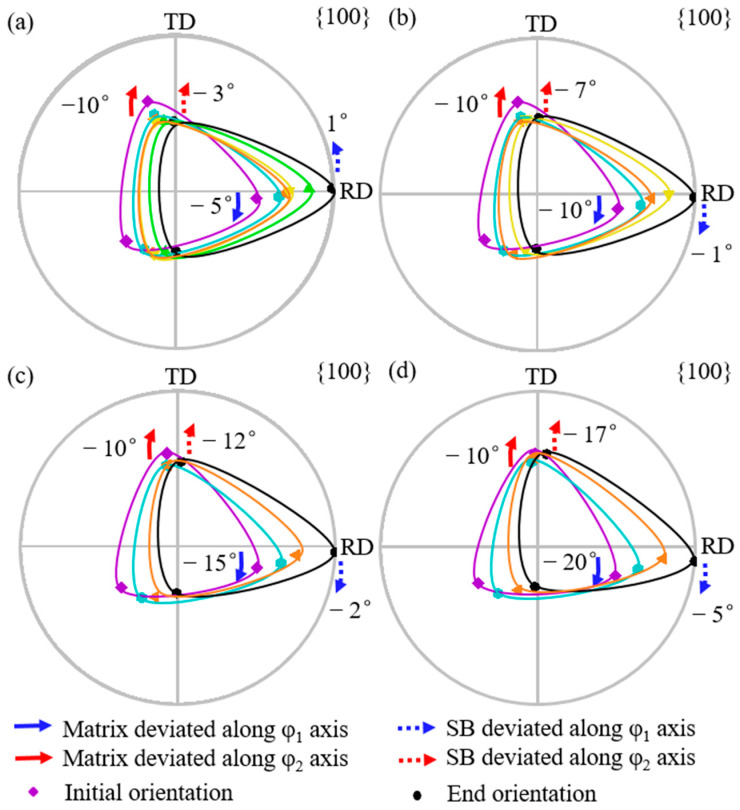
Simulated orientation rotation path of shear band in the matrix deviated by 10° along φ_2_ axis and 5°, 10°, 15°, and 20° along φ_1_ axis (**a**–**d**).

Figure 8 shows the simulated orientation rotation path of the shear band when the matrix is deviated by 10° along the φ_2_ axis with deviation by 5° to 20° (a–d) along the φ_1_ axis. For the matrix deviation along the φ_1_ axis from 5° to 20°, the shear band deviation along the φ_1_ axis, from 1° counterclockwise to 5° clockwise, is smaller than the matrix, while the shear band deviation along the φ_2_ axis increases from 3° to 17°. However, when the matrix is deviated by more than 10° along the φ_1_ axis, the shear band deviates at an angle greater along the φ_2_ axis than the matrix. The shear band orientation consistently exhibits a greater deviation along the φ_2_ axis compared with the φ_1_ axis.

**Figure 9:**
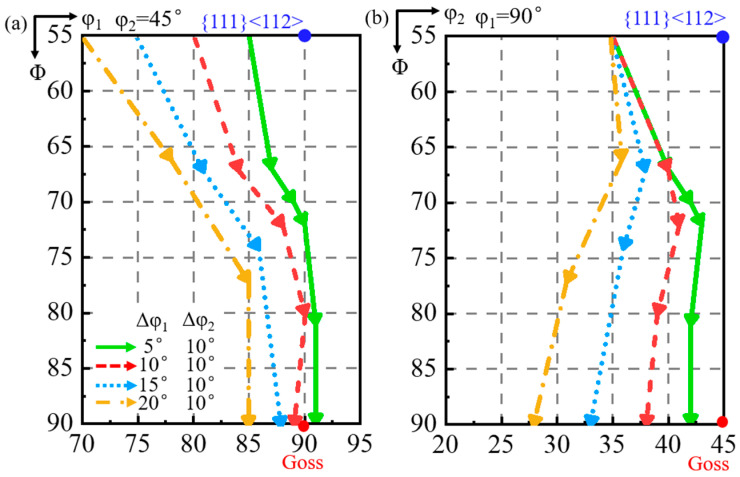
The projection of orientation rotation path of shear band in the matrix deviated by 10° along φ_2_ axis and 5°, 10°, 15°, and 20° along φ_1_ axis at constant (**a**) φ_2_ = 45° and (**b**) φ_1_ = 90° ODF sections.

As shown in Figure 9, the φ_1_ deviation of the shear band first decreases and then increases slightly during rotation. When the matrix deviation along the φ_1_ axis is 5°, the φ_1_ deviation of the shear band decreases to 0° and then increases in the opposite direction. The shear band deviation along the φ_2_ axis first decreases and then increases during rotation. When the matrix is deviated by more than 10° along the φ_1_ axis, the φ_2_ deviation of the shear band is progressively greater than the φ_2_ deviation of the matrix.

#### 3.2.2. 20° Deviation Along φ_2_ Axis

**Figure 10:**
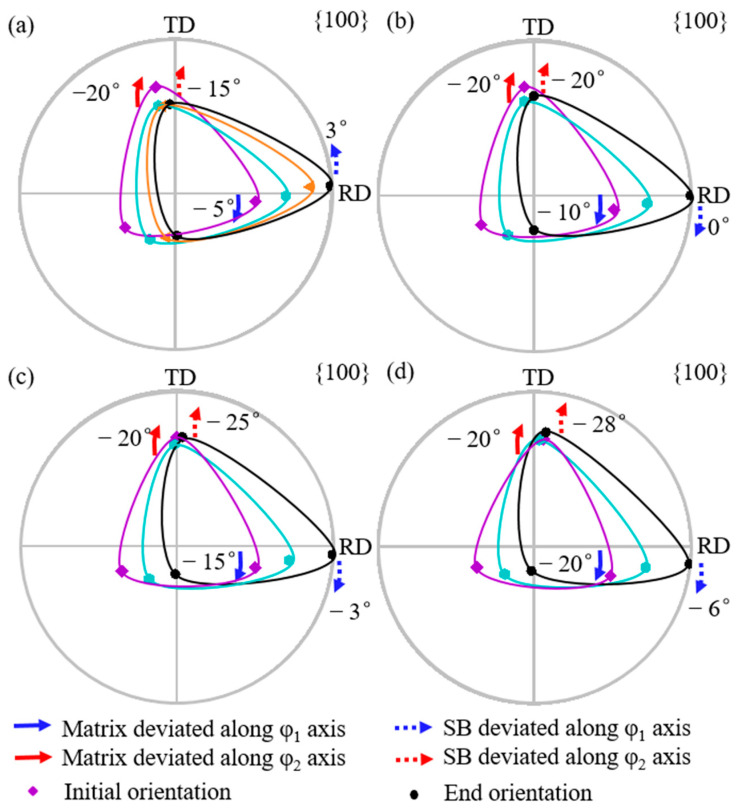
Simulated orientation rotation path of shear band in the matrix deviated by 20° along φ_2_ axis and 5°, 10°, 15°, and 20° along φ_1_ axis (**a**–**d**).

Figure 10 shows the simulated orientation rotation path of the shear band when the matrix is deviated by 20° deviation along the φ_2_ axis with deviation by 5° to 20° (a–d) along the φ_1_ axis. When the matrix deviation along the φ_1_ axis increases from 5° to 20°, the shear band deviation along the φ_1_ axis changes from 3° counterclockwise to 6° clockwise, while the shear band deviation along the φ_2_ axis increases from 15° to 28°. When the matrix is deviated by more than 5° along the φ_1_ axis, the shear band deviates at an angle greater than the matrix along the φ_2_ axis. The shear band consistently exhibits a larger deviation along the φ_2_ axis than the φ_1_ axis.

**Figure 11:**
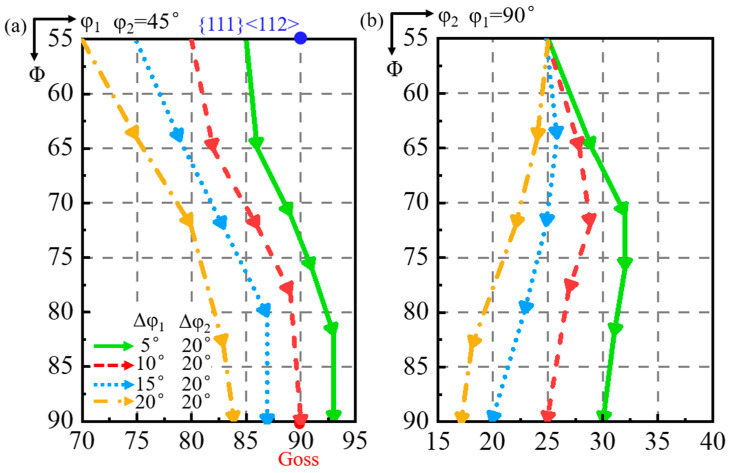
The projection of orientation rotation path of shear band in the matrix deviated by 20° along φ_2_ axis and 5°, 10°, 15°, and 20° along φ_1_ axis at constant (**a**) φ_2_ = 45° and (**b**) φ_1_ = 90° ODF sections.

Figure 11 shows the projection of the orientation rotation path of the shear band. When the φ_1_ deviation of the matrix is 5°, that of the shear band decreases to 0° and then increases in the opposite direction, while the φ_2_ deviation first decreases and then increases. When the matrix is deviated by more than 5° along the φ_1_ axis, the φ_2_ deviation of the shear band becomes progressively greater than the φ_2_ deviation of the matrix.

According to the above simulation, the orientation deviation type of the matrix evidently affects the rotation path of the shear band. When the matrix is uniaxially deviated from {111}<112> by 10° along the φ_1_ or φ_2_ axis, the φ_1_ deviation of the shear band decreases by 2°. In contrast, the φ_2_ deviation of the shear band increases by 4° when {111}<112> is deviated by 10° along both axes. When the matrix is uniaxially deviated by 20° along the φ_1_ or φ_2_ axis, the φ_1_ deviation of the shear band remains unchanged, whereas the φ_2_ deviation of the shear band increases by 18° when {111}<112> is deviated by 20° along both axes. This indicates that, compared with uniaxial deviation, the biaxial deviation of the matrix from {111}<112> results in either a decrease or no change in the φ_1_ deviation of the shear band, and the shear band deviation along the φ_2_ axis increases consistently.

### 3.3. Measurement of Shear Band Deviation

**Figure 12:**
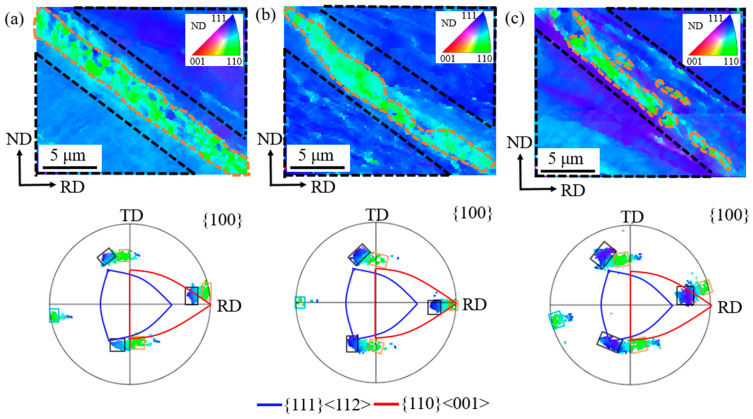
Orientation morphologies and {100} pole figures of shear band region 1 (**a**), region 2 (**b**) and region 3 (**c**).

Figure 12 presents the orientations and morphologies of three shear band regions in cold rolled grain-oriented silicon steel. The matrices and shear bands are outlined with black and orange dashed lines, respectively. In {100} pole figures, orientation points of the matrix and shear band are circled with black and orange boxes. In region 1, the matrix has an orientation at (81°, 60°, 49°) with a predominant φ1 uniaxial deviation from {111}<112>, and the shear band has an orientation at (82°, 90°,46°) with an 8° deviation from Goss. Compared with the matrix, the shear band deviation decreases by 1° along the φ1 axis and 3° along the φ2 axis. The simulated shear band orientation is located at (85°, 90°, 48°), deviated by 6° from Goss and 2° from the measurement.

In region 2, the matrix zone has an orientation focused at (89°, 62°, 41°), with a predominant φ_2_ uniaxial deviation from {111}<112>. The shear band zone has an orientation focused at (92°, 90°, 44°), with a 2° deviation from Goss. Relative to the matrix, the shear band deviation first decreases and then increases by 2° along the φ_1_ axis and decreases by 3° along the φ_2_ axis. The simulated shear band orientation based on matrix orientation is located at (91°, 90°, 44°), deviated by 1° from Goss and 1° from the measurement.

In region 3, the matrix has an orientation at (80°, 57°, 57°) with a biaxial deviation from {111}<112>. The shear band region has an orientation at (81°, 90°, 46°) with a 9° deviation from Goss. Compared with the matrix, the shear band deviation decreases by 1° along the φ_1_ axis and 11° along the φ_2_ axis. The simulated shear band orientation is located at (84°, 90°, 50°) with an 8° deviation from Goss, having only 1° difference from the measurement. This demonstrates that the present simulation effectively predicts the shear band orientation in the matrix deviated from {111}<112>.

## 4. Discussion

Based on the simulation and measurement, the shear band deviation from Goss along the φ_1_ and φ_2_ axes exhibits various dependencies on matrix orientation deviating from {111}<112>. The deviation angle change in the shear band in the rotation path can be attributed to the orientation rotation rate along the φ_1_ and φ_2_ axes [34,35].

### 4.1. Rotation Rate of Shear Band in Uniaxially Deviated Matrix

**Figure 13:**
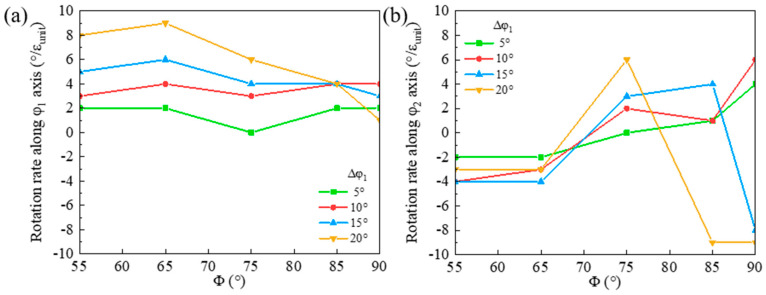
Variation of orientation rotation rate in shear band along (**a**) φ_1_ and (**b**) φ_2_ axes for {111}<112> matrix deviated along φ_1_ axis.

The orientation rotation rate morphologies of the shear band along the φ_1_ and φ_2_ axes in the deviated {111}<112> matrix along the φ_1_ axis are presented in Figure 13. The φ_1_ rotation rate decreases along the rotation path, and a larger φ_1_ deviation of the matrix corresponds to a notably higher rotation rate along the φ_1_ axis. This enables the φ_1_ angle to approach 90° with a decreased deviation along the φ_1_ axis. For the φ_1_ deviation of the matrix below 10°, the rotation rate of the shear band along the φ_2_ axis transits from negative to positive, so that the shear band deviation from Goss initially increases and then approaches Goss. However, when the φ_1_ deviation of the matrix exceeds 15°, the rotation rate of the shear band along the φ_2_ axis ultimately becomes negative again, and the shear band deviation along φ_2_ from Goss increases consequently.

**Figure 14:**
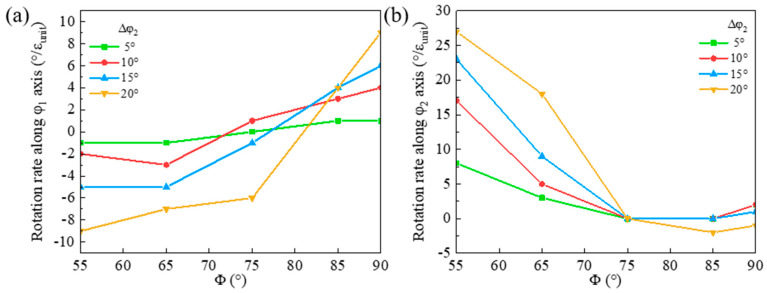
Variation of orientation rotation rate in shear band along (**a**) φ_1_ and (**b**) φ_2_ axes for {111}<112> matrix deviated along φ_2_ axis.

The orientation rotation rates of the shear band along the φ_1_ and φ_2_ axes for the {111}<112> matrix deviated along the φ_2_ axis are shown in Figure 14. A larger φ_2_ deviation of the matrix results in a higher negative rotation rate along the φ_1_ axis in the rotation path below Φ = 80° and a higher positive φ_1_ rotation rate in the range of Φ = 80~90°. Thus, the shear band deviation along the φ_1_ axis from Goss first increases and then slightly decreases. There is a large rotation rate of the shear band along the φ_2_ axis especially in the matrix with large deviation along the φ_2_ axis. However, when Φ > 75°, the rotation rate is relatively low, so the shear band deviation along the φ_2_ axis from Goss decreases during cold rolling. Furthermore, the larger negative rotation rate of the shear band in the matrix deviated along the φ_2_ axis leads to a larger shear band deviation from Goss compared with the case of the matrix deviated along the φ_1_ axis.

### 4.2. Rotation Rate of Shear Band in Biaxially Deviated Matrix

**Figure 15:**
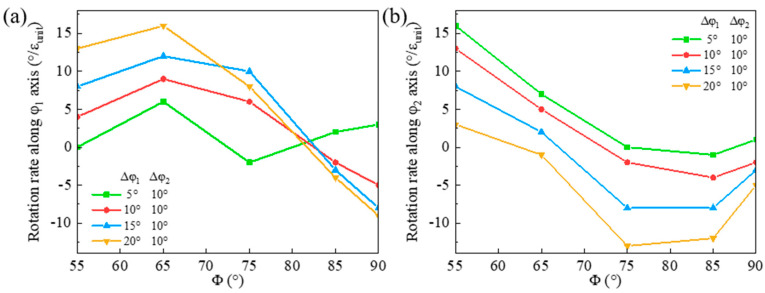
Variation of orientation rotation rate of shear band along (**a**) φ_1_ and (**b**) φ_2_ axis in (90°, 55°, 35°) matrix deviated along φ_1_ axis.

The orientation rotation rates of the shear band along the φ_1_ and φ_2_ axes in the (90°, 55°, 35°) matrix are shown in Figure 15. The absolute value of the rotation rate along the φ_1_ axis decreases along the rotation path. A larger matrix deviation along the φ_1_ axis results in a higher absolute rotation rate along the φ_1_ axis. However, the absolute rotation rate decreases more significantly as Φ increases, which is responsible for the decrease in φ_1_ deviation of the shear band. The rotation rate of the shear band along the φ_2_ axis also decreases during rotation and turns from positive to negative, so that the shear band orientation first rotates toward and then away from Goss. A larger matrix deviation along the φ_1_ axis corresponds to a lower rotation rate along the φ_2_ axis, leading to an ultimately increased φ_2_ deviation of the shear band. Additionally, the reduction in the rotation rate along the φ_2_ axis from positive to negative is more rapid than the φ_1_ axis, which leads to a more significant shear band deviation along the φ_2_ axis.

The orientation rotation rates of the shear band in (90°, 55°, 25°) matrix along the φ_1_ and φ_2_ axes are similar to the (90°, 55°, 35°) matrix. Compared with the matrix uniaxially deviated along the φ_1_ or φ_2_ axis, the shear band orientation in the matrix biaxially deviated along both axes has an larger negative rotation rate along the φ_2_ axis near Goss orientation. Consequently, the shear band has a larger deviation from Goss along the φ_2_ axis in the biaxially deviated {111}<112> matrix.

The crystal orientation always rotates to converge towards the orientation with higher stability or to diverge from the orientation with lower stability [36]. The deviation angle of the shear band along the φ_1_ axis decreases during rolling, indicating that Goss orientation exhibits a greater stability with respect to the φ_1_ axis deviation compared with the φ_2_ axis.

### 4.3. Applications and Limitations of the Model

The orientation deviation of the shear band leads to an orientation deviation of recrystallized Goss grains, and consequently influences the accuracy of secondary recrystallization Goss texture [37,38]. The shear band deviation along the φ1 axis results in the secondary recrystallization texture misaligning with <001> easy magnetization axis along RD, thereby reducing the magnetic properties of the final product. The shear band deviation along the φ_2_ axis results in the deviated <110> from ND after secondary recrystallization [39], which also degrades the magnetic properties.

In industrial production, parameters such as carbon content and rolling temperature are employed to regulate the activation and number of shear bands [40]. However, the shear band orientation basically relies on matrix orientation, which is more involved in the production process of grain-oriented silicon steel. In the present study, the crystal plasticity simulation with a 2D inclined angle of the shear band depending on matrix orientation can represent the general orientation rotation path in the shear band for different matrices deviated from {111}<112> in actual industrial processes.

The VPSC model and this study assume a homogenized deformation of the shear band, which may overlook the local heterogeneities or variations in shear bands that can influence general orientation diffusion behavior, especially at a smaller scale.

## 5. Conclusion

Shear band orientation in deviated {111}<112> matrix was investigated using crystal plasticity simulation by introducing a two-dimensional inclination of the shear band plane-dependent matrix orientation as well as experimental measurement.

(1) In uniaxially deviated {111}<112> matrix along the φ_1_ (or φ_2_) axis, the shear band deviation in the φ_1_ (or φ_2_) axis from Goss decreases during rotation, while the deviation in the φ_2_ (or φ_1_) axis either first increases and then decreases or monotonically increases. The larger negative orientation rotation rate of the shear band in the matrix deviated along the φ_2_ axis results in the larger shear band deviation from Goss compared with the matrix deviated along the φ_1_ axis.
(2) In the {111}<112> matrix deviated along both φ_1_ and φ_2_ axes, the shear band deviation along φ_1_ decreases throughout the rotation process, while the deviation along φ_2_ first decreases and then increases. The rapidly decreased rotation rate along the φ_2_ axis is responsible for the larger shear band deviation from Goss along the φ_2_ axis than the φ_1_ axis.
(3) Compared with uniaxially deviated {111}<112> matrix, the biaxially deviated matrix can have a reduced or unchanged deviation along the φ_1_ axis and an increased deviation along the φ_2_ axis, resulting in a larger overall deviation in the shear band from Goss. This difference arises from the enhanced rotation rate along the φ_1_ axis and the decreased rotation rate along the φ_2_ axis under biaxial deviation of the matrix.
(4) The simulation method used is capable of predicting the general rotation path in the shear band, and can be extended to other BCC metallic materials. However, the present simulation is based on the assumptions that a simple shear strain occurs in the shear band and the orientation rotation in the shear band is uniform. Further simulation work is needed to investigate the orientation dispersion in the shear band due to the microscopic strain heterogeneity.

## Data Availability

The raw/processed data can be made available by the corresponding author upon reasonable request.
